# Experiences of an earthquake during pregnancy, antenatal mental health and infants’ birthweight in Bhaktapur District, Nepal, 2015: a population-based cohort study

**DOI:** 10.1186/s12884-020-03086-5

**Published:** 2020-07-20

**Authors:** Goma Kumari Khatri, Thach Duc Tran, Sushil Baral, Jane Fisher

**Affiliations:** 1 School of Public Health and Preventive Medicine, Monash University, Level 4, 553 St Kilda Rd, Melbourne, Victoria 3004 Australia; 2 HERD International, Kathmandu, 44600 Nepal

**Keywords:** Birthweight, Earthquake experiences, Antenatal, Common mental disorders

## Abstract

**Background:**

Infant birthweight is an important public health indicator that is a proxy of maternal and baby’s health. Earthquakes can cause acute distress, but can also contribute to chronic stress through long-term disruptions to social, economic and domestic circumstances. The aims of this study were to examine the direct effect of earthquake experiences on the birthweight of infants of women who experienced the 2015 Nepal Earthquakes during pregnancy and whether mental health mediated this relationship.

**Methods:**

This is a prospective, population-based cohort study. Pregnant women in Bhaktapur District, Nepal who had experienced the 2015 earthquakes after conception were recruited. Baseline data were collected in structured individual interviews. Follow up was a short telephone interview two weeks after the baby’s birth. Infant birthweight recorded on the hospital-issued birth certificate. The direct effect of earthquake experiences on birthweight and the indirect effect via antenatal common mental disorders (CMDs) were tested using Sobel tests simultaneously.

**Results:**

In total 497/498 women contributed baseline data and 469 (94.4%) women also provided the birth weight of their infants. In total 6% of infants had low birth weight (< 2.5 kg). The earthquake experiences did not have a significant direct effect on birthweight (*p* = 0.116). However, the effect of earthquake experiences had a significant indirect effect on infant birthweight via CMDs. When 15 covariates were controlled, women who had moderate to severe earthquake experiences had an increase of 1.58 scores of antenatal CMD symptoms that resulted in their babies being 20.50 g lighter than those who had low earthquake experiences (*p* = 0.026).

**Conclusions:**

The findings from this study indicate that the relationship is mediated by the mental health of women during pregnancy. Data demonstrate that the mental health of women who are pregnant should be prioritised in post-disaster management not only because of the burden experienced by women but also because of the risk for the growth and development of their babies.

## Background

Infant birthweight is an important public health indicator. It reflects maternal health as well as a predictor of the health and development of the baby [1]. Worldwide, it is estimated that low birthweight (LBW), usually defined as being born weighing less than 2500 g, contributes to up to 80% of all neonatal deaths [2]. It is estimated that about 96.5% of infants born with LBW are living in resource-constrained low-income countries [2]. Finding from a national survey in Nepal revealed that LBW of infants based on the reported birth weight by mothers who had a live birth in the five years prior to the survey was 12.3%. About 5 and 12% of the mothers reported that their babies were small and very small at birth respectively [1]. Babies with LBW who survive, are at higher risk of compromised growth and development in their subsequent lives [3].

Multiple causative factors are associated with LBW, but maternal health is a well-established determinant. While poor maternal nutrition, and chronic or acute stresses during pregnancy independently increase the likelihood of LBW, these may interact with each other in ways that amplify the risk of LBW [2, 4]. These causative factors are highly prevalent in low-income settings [2, 5, 6].

In Nepal, Sharma et al. [7] conducted a case-control study in a tertiary hospital to identify determinants of LBW. They found that women who had a history of premature birth, had not been able to consume nutritious food during pregnancy, and were of younger (< 20 years) age had a significantly higher risk of giving birth to a LBW infant [7]. We are not aware of any study in Nepal that has examined and reported on associations between pregnancy mental health and infant birthweight.

Earthquakes cause acute distress, but can also contribute to chronic stress through long-term disruptions to social, economic and domestic circumstances [8–10]. Our study [11] was conducted after the 2015 Nepal earthquakes and reported that 21.9% of participants had Edinburgh Postnatal Depression Scale (EPDS) scores> 12, and another 17.1% scored 10–12 indicating a high prevalence of clinically-significant common mental disorders (CMDs) symptoms, which include anxiety, depressive, adjustment, and somatoform disorders [12]. This is a higher prevalence than those reported in a systematic review and meta-analysis (about 16%) from low and lower-middle-income countries in general circumstances [5]. Another study by Kane et al. [13] was conducted in the same earthquake context among the general population. The study reported that 34.3% experienced depressive symptoms, 33.8% anxiety and 5.2% had PTSD symptoms. The study found that women were more likely to experience depression, anxiety and PTSD then men [13].

In general circumstances, antenatal CMDs appear to increase the risk of LBW [6, 14]. Grote et al. [6] conducted a systematic review of eleven studies reported from ‘the United States’ and ‘non-United States countries’ and found that relative risk of LBW was significantly higher among women who had ‘antenatal depression’ compared to those who had not. The study also reported that LBW was significantly higher in ‘non-United States’ countries compared to those in ‘the United States’ [6].

There have been seven studies that reported that women’s experiences of an earthquake might increase the risk of giving birth to LBW of their infants. Among them, six [15–20] were from middle-and high-income countries and only one [21] from a low-income country. All were audits of birth records or secondary analyses of existing data. None investigated directly, the relationships among women’s experiences of the earthquake while pregnant, their mental health and its relationship with the birthweight of their infants.

There is to date no study examining the possible pathways of the effect of earthquakes on birth outcomes, but this is of central importance for effective post-disaster responses. Antenatal mental health that can be compromised by experiencing a natural disaster and might play a mediating role in this relationship. The aims of this study were to examine the direct effect of earthquake experiences on the birthweight of infants of women who experienced the 2015 Nepal Earthquakes during pregnancy and whether mental health mediated this relationship.

## Methods

### Study design

A prospective, population-based study of a cohort of women who were pregnant and had experienced the 2015 Earthquakes since conceiving. It included a baseline survey in late pregnancy and a follow-up interview conducted two weeks after birth.

### Study setting

Data were collected in Bhaktapur District from October 2015 to April 2016. Bhaktapur is one of the 14 of 77 districts in Nepal that were severely affected by the April 2015 Earthquakes which were measured at 7.8 on the Richter Scale [22]. Bhaktapur has six sub-administrative divisions that include rural and urban communities. It also includes a UNESCO-designated World Heritage Site of major cultural, historical, and religious significance.

### Conceptual framework

**Fig. 1:**
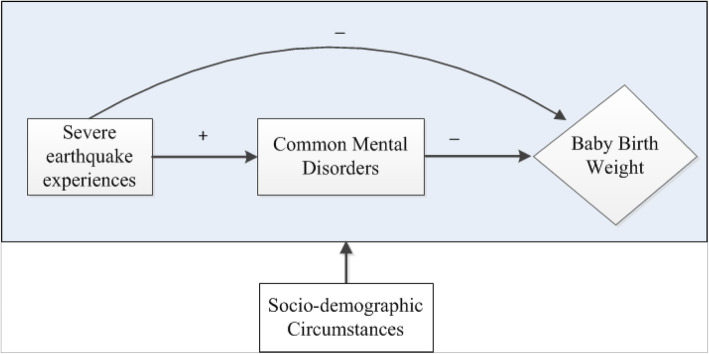
Conceptual framework. **Source:** Khatri, GK (2019) [23]. Note. *Arrow signs show the postulated direction of association among variables. Plus (+) sign indicates positive and minus (−) sign indicates a negative association between variables*

Conceptual framework in (Fig. 1) shows the proposed direction of associations among study variables.

### Participants

The sample size was calculated to test the indirect effect of earthquake experiences on birthweight via antenatal CMD symptoms with a study power of 80% and an alpha error of 0.05 (5% level of significance*)*. In total 480 women were required to identify Cohen’s effect ≥0.14 of earthquake experiences on CMD symptoms and ≥ 0.26 of CMD symptoms on birthweight, allowing for attrition of 20% [24].

All women were invited to participate in the study if they met the following criteria: (1) to be aged at least 15 years, (2) living in Bhaktapur District, and (3) pregnant during the 2015 Earthquakes. Pregnant women were excluded if they: (1) had a multifetal pregnancy, or (2) had a cognitive disability, or (3) were local but outside Bhaktapur at the time of the major 2015 earthquakes, or (4) moved (migrated) to Bhaktapur to live after the major earthquakes.

Those who contributed baseline data were invited again to participate in the follow-up interview. Women who had a stillbirth and those who did not measure the weight of their babies at birth were excluded from analyses on birthweight.

### Public involvement

Members of the local community were included in diverse ways. Local bilingual health experts contributed to the translation of the interview schedules to Nepali, cultural verification and back translation to English. Twelve pregnant women are meeting inclusion criteria apart from living in Bhaktapur pilot-tested the schedules for acceptability and comprehensibility. Health workers, community leaders, elderly women, female community health volunteers, and staff of the Bhaktapur District Women’s Development Office identified eligible pregnant women. HERD International, a local research organisation, partnered in conducting this study.

### Primary data sources

The primary data source was a structured interview schedule, which included standardised tools and study-specific questions (Table 1, Questionnaires).

**Table 1:** Primary data sources and coding

Interview time	Variables	Primary data sources and coding
	birth outcome	
Follow up	**Birthweight**	In Nepal, a birth certificate is provided to each mother who has given birth in a medical facility. It records the date and time of the birth and the baby’s birthweight. Women were asked to report the birth weight of their babies from the birth certificate. Women who gave birth at home and did not have a birth certificate but where a skilled person had measured birth weight using a standard scale were also asked to report the weight. Those who did not measure baby birth weight were not able to be included in analyses. The weight in grams was used as a continuous variable to examine an association among covariates. Birth weight was also categorized into two groups: ≥ 2.5 kg deemed to be normal and < 2.5 kg as low birth weight.
	Mediating variable	
Baseline	**Symptoms of Common Mental Disorders (CMDs) during pregnancy**	The Edinburgh Postnatal Depression Scale-Nepali version (EPDS-N) comprises 10 items, scored from 0 to 3 with a total score of 0 to 30. This version has been formally validated against Diagnostic and Statistical Manual (DSM) IV diagnostic criteria among women who had recently given birth in Nepal: Sensitivity 68.43%; Specificity 93.80%; Positive Predictive Value 65% and Negative Predictive Value 94.64 [31]. The EPDS total score was used as a continuous variable to examine a mediation effect. We also reported proportions of the scores: < 9, 10 to 12 and ≥ 13 to enable comparison between groups.
	Independent variables	
Baseline	**Earthquake experiences**	Study-specific questions adapted from previous studies [9, 20] collecting information about 17 items: where they are living now (1 item); property damage (3 items); impact on daily life including basic needs (6 items); being injured/trapped (2 items); witnessing injury/people trapped/death (4 items), intensity of fear experienced during the earthquake (1 item). Details of these items have reported elsewhere [11]. Each item was scored 0 (not experienced) to 1 (experienced) and summed to create a total score. The score ranged from 0 to 17. The total score was grouped in tertle, and low tertile was labelled as low, and middle and upper tertile labelled as middle/high earthquake experiences.
	Covariates	
Baseline and follow up	**Length of gestation at birth**	Date of the baby’s birth and the date of the last menstrual period were recorded to calculate the length of gestation at birth. At baseline, we asked participants about their date of last menstrual period. In the follow-up interview, date of baby birth was recorded. The last date of menstruation was subtracted from date of baby birth and divided by seven to calculate the total weeks as the length of gestation at birth. We used weeks as a continuous variable to examine an association with other variables.
Baseline	**Socio-demographic characteristics**	Study-specific questions adapted from our prior research [39] to ascertain women’s age, highest educational level, employment, consume chewing tobacco/smoking, and alcohol, household economics, family structure, and husband’s education, employment, consumption of chewing tobacco/smoking, and alcohol. Household economics was calculated based on household characteristics and durable assets using the World Bank method [39, 40].
Baseline	**Woman’s body mass index**	Woman’s weight was measured using a portable digital weighing scale. Height was measured against the smooth but hard wall. Height and weight were used to calculate body mass index using international formula (weight/height^2^). It was used as a continuous variable to examine its associations with other covariates.
Baseline	**Reproductive health**	Study-specific questions adapted from our prior research [39] about gravidity, parity, prior spontaneous/induced abortions, pregnancy intention, taking iron tablets during current pregnancy, foetal health, sex of index foetus and a number of children.
Baseline	**History of psychiatric illness**	Single yes/no question was used to assess if the participant had been diagnosed with or treated for any psychiatric illness within last year.
	**Intimate partner relationship**	
Baseline	Quality of relationship with the current intimate partner	The Relationship Assessment Scale (RAS) comprises 7 items scored from 1 to 5 and total scored from 7 to 35. Mean inter-item correlation of the scale is 0.49; test-retest reliability (among undergraduate students in USA) is 0.85 and high correlation with Dyadic Adjustment Scale [41] (0.86 to .88). Examples of items are: ‘How well does your partner meet your needs? In general, how satisfied are you with your relationship? How much do you love your partner?’ Higher scores indicate a better quality of the relationship [42].
Baseline	Experiences of intimate partner violence	Items from the World Health Organization Multi-Country Study on Women’s Health and Violence Against Women Questionnaire, including lifetime experiences of controlling behaviour (7 items), emotional (4 items), physical (6 items), and sexual (3 items) violence by the current intimate partner [43]. Any lifetime experiences of intimate partner violence calculated combining any of four sub-types of violence.
Baseline	Practical and emotional support specific to the earthquakes	Study-specific questions adapted from previous studies [10, 20]. Participants were asked whether they received any support including shelter, food, clothes, informal emotional and professional psychological support and rescue from the hazardous place during/after earthquakes by any individual or any governmental and non-governmental organization.

### Procedure

Participants were recruited from all Bhaktapur sub-administrative divisions. Potential participants were identified in three ways: (i) together, the district public health office, district public health nurses, community health centre staff and female community health volunteers (FCHVs) created a list of eligible pregnant women (ii) The Women and Children Development district officers identified eligible women who had not been listed (iii) formal and informal local community leaders, elderly women and pregnant women were asked to identify potential participants in their villages. With the address given in the list of eligible women, we visited their home to inform them about the study and invite to participate in the study.

There were two waves of data collection: one during pregnancy and the other two weeks after women had given birth.

Potential participants were informed about the study by giving an explanatory statement. The statement was read by a researcher or a family member to those who could not read it. Participants were assured that their privacy and confidentiality would be maintained strictly. Women who were willing to participate gave consent by signing the form or providing a thumbprint.

When a participant gave consent, she was invited to complete an interview in person for the first wave of data collection (baseline). All interviews were conducted in Nepali in private rooms at local health centres or, rarely, in a private space at their homes.

Six trained and female interviewers who had experience in data collection for other research projects managed by HERD International conducted the interviews following training from and with supervision from bilingual co-investigator (GKK).

The second interview (follow-up) was a very short telephone interview two weeks after childbirth to ask about the date, place and mode of birth, and the baby’s birthweight. All these interviews were conducted by GKK.

### Data management and statistical analysis

Data were collected on paper forms and double-entered using Epidata version 3.1 computer software [25] at HERD Nepal. Data analyses were performed using Stata version 14 [26]. Descriptive analyses were used to compare participants’ characteristics in the group who provided complete data (which could be included in the final analyses) with those of participants who provided incomplete data (which could not be included) and between groups with babies of different birthweights. Fisher exact test for categorical and Mann-Whitney test for continuous variables were used to test socio-demographic differences between the groups were and were not included in the analyses.

The direct effect of earthquake experiences on birthweight and the indirect effect via EPDS total score were tested using simultaneous Sobel tests [27, 28]. According to Pek and Hoyle [28], we assumed that women’s mental health problems (EPDS total score) mediated the effect of earthquake experiences on birthweights. In this analysis, birth weight (in grams) was the primary outcome, the exposure variable was earthquake experiences, and EPDS total score was a mediating variable [28]. This analysis controlled for 15 covariates that were selected based on the results of bivariable analyses and previous studies.

Bivariate analyses were conducted using the chi-square test or regression to select potential covariates that should be included in the final model of the mediation analysis. Covariates were selected on the basis that there were significant differences at *p* ≤ 0.1. However, some covariates such as household wealth, intimate partner violence and education levels were included in the final model even though they were not significant because they are established risk factors for low birthweight [29, 30].

In the Sobel test, three different multiple linear regression models were performed simultaneously. The first model predicted birthweight from earthquake experiences and other covariates but without EPDS scores. The second model predicted EPDS scores from earthquake experiences and other covariates. The final model predicted birthweight from all variables including earthquake experiences, EPDS scores and other covariates. Finally, the test results were summarized including the estimations and significant tests of the direct effect of the earthquake experiences on birthweight; the indirect effect of the earthquake experiences on birthweight via antenatal CMD symptoms, and the total effect of the earthquake experiences on birthweight.

In order to examine the interaction effect between the exposure (earthquake experiences) and mediator (depressive symptoms) on birthweight and the confounding effect of depressive symptoms on the effect of earthquake experiences on birthweight, we conducted multivariate regression analyses stratified by having depressive symptoms or not. The criterion for not having clinically significant depressive symptoms was an EPDS score ≤ 12. The criterion for having clinically significant depressive symptoms was an EPDS score ≥ 13. These criteria were based on a formal validation of the EPDS among women who had recently given birth in Nepal [31]. The multivariate regression predicting birthweight included all covariates that had been included in the mediation analysis.

## Results

### Sample

**Fig. 2:**
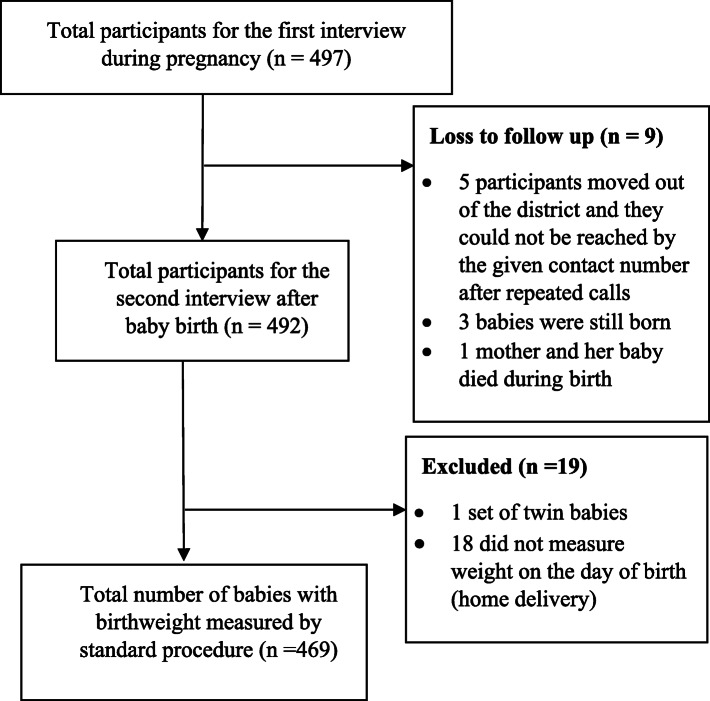
Flow diagram of participants able to provide data about their baby’s birth weight. Note. *n = number*

In total 497/498 eligible pregnant women were recruited and contributed baseline data. All gave consent to be followed up, and 492 women participated in the follow-up interview. Altogether 469 (94.4%) were able to provide the baby’s weight at birth and were included in the analyses (Fig. 2).

### Participants’ characteristics

A comparison of the characteristics of participants whose data could and could not be included in the final analyses is presented in Table 2. Compared to the group who could be included, the group who were not included had significantly younger age, lower body mass index, no formal or primary education, more women consumed chewing tobacco/smoking, and lower household wealth. In addition, they were more likely to have a no-formal educated partner, experienced emotional and physical/sexual violence perpetrated by an intimate partner, had an inconvenient/unwanted pregnancy, lower ANC check-ups and iron tablet intake.

**Table 2:** Characteristics of participants at the baseline interview

Characteristics	Included (*n* = 469)	Not included (*n* = 28)	*p*-value
Socio-demographic characteristics			
Age (years), mean (SD)	26.5 (4.8)	24.9 (5.2)	0.05
Body mass index during late pregnancy, mean (SD)	27.1 (3.8)	25.4 (3.8)	0.01
Education, n %			< 0.001
No formal/ primary education	86 (18.3)	17 (60.7)	
Secondary and above education	383 (81.7)	11 (39.3)	
Having income-generating work, n %	152 (32.4)	8 (28.6)	0.8
Consume chewing tobacco/smoking, n %	12 (2.6)	7 (25.0)	< 0.001
Alcohol consumption, n %	118 (25.2)	7 (25.0)	1.0
Family structure, n %			0.3
Joint/extended family	234 (49.9)	11 (39.3)	
Nuclear family	235 (50.1)	17 (60.7)	
Education of partners, n %			< 0.001
No formal/ primary education	51 (10.9)	13 (46.4)	
secondary and above education	418 (89.1)	15 (53.6)	
Partners with income-generating work, n %	437 (93.2)	27 (96.4)	1.0
Consume chewing tobacco/smoking by partner, n %	157 (33.5)	20 (71.4)	< 0.001
Alcohol consumption by partner, n %	239 (51.0)	19 (67.9)	0.1
Household wealth, mean (SD)	.09 (2.03)	−1.53 (1.66)	< 0.001
Intimate partner relationship			
Quality of relationship with an intimate partner, n %			0.002
Least optimal (< 28 RAS score)	105 (22.4)	14 (50.0)	
Optimal (> 28 RAS score)	364 (77.6)	14 (50.0)	
Any lifetime experience of intimate partner controlling behaviour, n %	163 (34.8)	15 (50.0)	0.1
Any lifetime experience of intimate partner emotional violence, n %	191 (40.7)	17 (60.7)	0.05
Any lifetime experience of intimate partner physical and or sexual violence, n %	109 (23.2)	16 (57.1)	< 0.001
Any lifetime experiences of any intimate partner violence, n %	270 (57.6)	20 (71.4)	0.2
Reproductive characteristics			
History of pregnancy, n %			
Nulliparous	211 (45.0)	9 (32.1)	0.2
Two or more pregnancies	258 (55.0)	19 (67.9)	
History of miscarriage/abortion, n %	85 (18.1)	7 (25.0)	0.3
Taking iron tablet during current pregnancy, n %	449 (95.7)	20 (71.4)	< 0.001
The good foetal health, n %	447 (95.3)	24 (85.7)	0.05
Unwelcome/inconvenient pregnancy, n %	61 (13.0)	10 (35.7)	0.003
Attended antenatal check-up for current pregnancy	466 (99.4)	25 (89.3)	0.003
Number of the antenatal visit for current pregnancy			< 0.001
none	3 (0.6)	3 (10.7)	
once	8 (1.7)	2 (7.1)	
twice	15 (3.2)	5 (17.9)	
Three times	33 (7.0)	5 (17.9)	
Four or more times	410 (87.4)	13 (46.4)	
Sex of index foetus			0.7
Boy	262 (55.9)	14 (60.9)	
Girl	207 (44.1)	9 (39.1)	
Number of children, n %			0.6
None	246 (52.5)	13 (46.4)	
One or more	223 (47.5)	15 (53.6)	
Length of gestation at baby birth (weeks), mean (SD)	39.9 (1.5)	39.1 (2.5)	0.2
Earthquake experiences, mean (SD)	3.3 (1.3)	3.6 (1.3)	0.2
Having practical/emotional social support, n %	348 (74.2)	18 (64.2)	0.3
EPDS, mean (SD)	8.0 (5.1)	10.6 (6.7)	0.06
EPDS total score categories, n %			0.2
< 9 score	289 (61.6)	14 (50.0)	
10 to 12 score	81 (17.3)	4 (14.3)	
≥ 13 score	99 (21.1)	10 (35.7)	

Note. *‘Included’ means data included for final analysis; ‘not included’ means data not included for final analysis (loss to follow up)*

n = number; % = percentage; SD = standard deviation; EPDS = Edinburgh Postnatal Depression Scale

No women reported a history of psychiatric illness.

### Birth weight

Birthweight ranged from 1800 g to 5000 g. Mean birthweight was 3045.3 ± 447.8 g. About 6% (5.8%) women reported birthweight less than 2500 g. Most women reported a rounded birthweight: 13.65% reported 3000 g; 11.51% reported 3500 g; 9.81% reported 2500 g; 8.10% reported 2700 g; 6.18% reported 2800 g and about 5% in each weight category reported 2900 g, 3000 g, 3100 g and 3200 g.

### Association among earthquake experiences, antenatal CMDs symptoms and birth weight

**Fig. 3:**
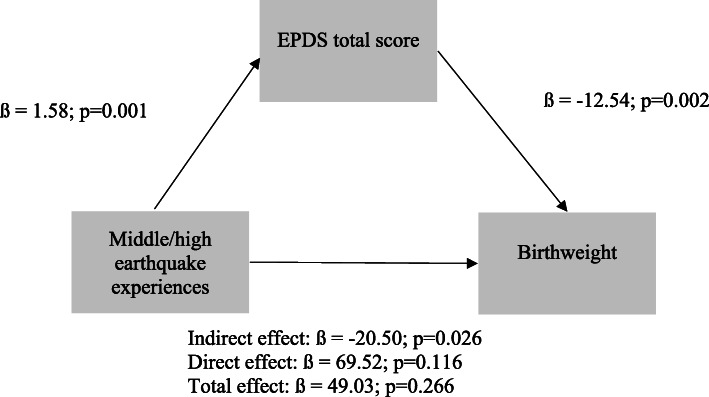
Key findings of the Sobel test of the direct effect of earthquake experiences on birthweight and the indirect effect mediated by EPDS total score. Note*. Single-headed solid arrows show the direction of association among variables. EPDS = Edinburgh Postnatal Depression Scale; ß = linear regression coefficient*

The main findings of the Sobel test analysis are presented in Fig. 3, and more detailed findings are presented in Tables S1, S2, S3 and S4. The results showed that earthquake experiences did not have a significant direct effect on birth weight. Compared to low experiences of the earthquakes, women who had middle or high earthquake experiences had higher EPDS scores. Women who had higher EPDS scores had babies with lower birthweights.

Antenatal CMDs symptoms significantly mediated the effect of earthquake experiences on baby birthweight (Fig. 3). This means that while controlling for all covariates, women who had moderate to high earthquake experiences had an increase of 1.58 points in the EPDS scores that resulted in a 20.50 g reduction in their infants’ birth weight compared to those who had low earthquake experiences.

The multivariate regression analyses predicting birthweight stratified by having or not having clinically significant depressive symptoms revealed that moderate to high experiences of the earthquake had no significant effect on birthweight in the absence of depressive symptoms (adjusted coefficient = 52.26, *p* = 0.29) or in the presence of depressive symptoms (adjusted coefficient = 171.94, *p* = 0.10).

Among the covariates, body mass index of women during advanced pregnancy and length of gestational age at birth were also associated consistently and independently with birthweight (Table S1, S3).

## Discussion

To our knowledge, this is the world’s first study in a low-income country to examine the relationship between pregnant women’s experiences of an earthquake and the birthweight of their infants and whether antenatal CMD symptoms mediate the effect of earthquake experiences on baby birthweight. Women who had moderate or high earthquake experiences gave birth to babies who weighed 20.50 g less compared to women who had low earthquake experiences via antenatal CMD symptoms. Independent of other covariates, women who had higher CMD symptoms gave birth to babies with significantly lower birthweight compared to women with lower CMD symptoms.

There is no study examining the direct and indirect effect of earthquake experiences on birth weight simultaneously to compare our findings. However, the findings of our study are in line with the findings of audits of hospital records that babies born after earthquakes had lower birthweight compared to those who born before the earthquakes [16, 18–21] and indicate that experiencing an earthquake while pregnant can adversely affect birth outcomes. Further, our study findings are in line with previous investigations of the relationship between antenatal CMDs and baby birthweight in general circumstances, which have found a significant negative association [6, 14]. One possible explanation for this is that earthquake experiences may increase stress in pregnant women that negatively affect the growth and development of their foetus rather than there being a direct effect of earthquake experiences on the foetus [6, 32].

About 6% of women reported that their babies had LBW (< 2500 g), but a further 10% of women reported a rounded Fig. 2500 g. It is common practice for rounding up to occur and so it is likely that almost 16% of babies who had been exposed in utero to the earthquake were of LBW.

Our study reported a higher prevalence of LBW than those of other studies conducted in an earthquake context. For instance, Tan et al. [19] audited birthweight of babies from hospital records before and after the 2008 Wenchuan earthquake in China. They found that 5.01% of babies born after the earthquake had LBW (≤2500 g) which was significantly higher than those were born before the earthquake (3.72%). China is a middle-income country where the rate of LBW is much lower than in Nepal in general circumstances too [2]. Women in China may receive better care and services in post-earthquake management than those of Nepali women that may affect the difference of baby birthweight.

In Nepal context, our study reported a higher prevalence of LBW than that reported in the recent Nepal Demographic and Health Survey (NDHS) 2016 (12% baby had LBW < 2500 g) [1]. Our study site was in Bhaktapur district, which was relatively well resourced, with more accessible health services than in other districts in Nepal. The earthquake created a unique circumstance. There were anecdotal records from hospital staff that women heard informally that earthquakes might have a negative impact on foetal health and growth and ‘many women’ sought termination of their pregnancies. Women who elected to continue their pregnancies were reported to be cautious about foetal wellbeing, and they presented for antenatal check-ups (Table 2) at a much higher rate than the national average [1]. Despite this healthcare, LBW was still very high.

This study confirms previous findings [2, 4] that maternal body mass index is associated significantly with baby birthweight. While malnourishment among women in reproductive age in Nepal is high, women rarely consume additional nutritious food during pregnancy [1, 7]. According to NDHS 2016, 17.3% of women of reproductive age had a low body mass index (< 18.5 kg/m^2^), an indicator of undernutrition [1]. In Nepal, women, generally feed other family members first and then they consume any remaining food at the end of the meal. This practice applies to pregnant women too. Even though there are different nutritional policies and programs to combat nutritional problems [1], there is no policy or practise to ensure specific food security for pregnant women in post-earthquake management in Nepal [33]. It was reported that nutritious food was distributed to pregnant women immediately after the earthquake, but the report did not specify what nutritious food and what number of women benefited. The report did not provide details about who benefited from the supply: all women or only women in urban or in rural areas and how long the supplementation was sustained for [34, 35].

In contrast to the national scenario, only two participants had a low body mass index (< 18.5 kg/m^2^) in this study. Most of the study participants reported that they had received advice about nutrition and diet (96.2%) and took iron tablets (94.4%) during the current pregnancy. This could be related to the unique circumstance of the earthquake that this cohort of women may receive special attention to protect their foetus. When we used body mass index as a continuous variable to predict birthweight, it showed that with the increment of body mass index, birthweight of baby increased significantly and independently.

In contrast to a previous study [29], our study did not find household wealth to be a risk factor. The household wealth of this sample was relatively homogeneous, and there may have been insufficient variance to detect differences in birthweight between households with higher and lower wealth. A household wealth of women who were ‘included’ and were ‘not included’ in final analyses was significantly different. This suggests that ‘not included’ women were even more likely than those who were included to have a baby with LBW and that our findings were more likely to have been under- than over-estimates of the impact of the earthquake on birthweight.

Our study reported a small number of women (4.1%) had a preterm birth (< 37 weeks). This could be because we missed the group at high risk of preterm birth or who sought an abortion before we approached them since the data collected six months after the major earthquake. Because of the small number of preterm birth, length of gestation at birth was used as a continuous variable to predict birthweight. Consistent with previous studies [2, 7, 36], our data revealed that the higher the length of gestation at birth, the higher the weight of babies.

### Strengths

The study was a population-based prospective study. Population-based studies permit the findings to be generalised to equivalent settings. We were able to do a postpartum follow-up as a prospective study. Prospective investigations are able to ascertain causal relationships including, in this study, between earthquake experiences, pregnant women’s mental health and birthweight. This is a robust design for mediation analyses [28]. The study was adequately powered to detect the direct and indirect effects of the earthquake on birthweight.

There were very high recruitment and retention fractions. Data were collected in individual interviews which enabled women with low literacy to participate. We controlled for other known risk and protective factors for baby birthweight and antenatal CMD symptoms to control for potential confounding effects.

### Limitations

We acknowledge the limitation of the study that baby birthweight was based on the birth certificates provided by health institutions rather than being measured by the research team. Most birthweights were recorded on birth certificates as figures that had been rounded up; it is possible that the percentage of LBW was underestimated. For this reason, we used birthweight as a continuous measure in outcome analyses. The earthquake experience measurement scale was adapted from one used in previous studies and was carefully pilot tested, but it was not standardised or locally validated.

In Nepal, menstruation is considered unclean, and it is culturally prohibited for women to attend everyday religious observations or sacred ceremonies or to go into the home kitchen or to prepare food when they are menstruating. These are publicly observable circumstances and readily linked to specific ceremonies or events, and so it is easy for women in Nepal to recall the first day of the last menstrual period. It is generally believed that they rarely recall the date inaccurately. Nevertheless, we acknowledge that the date of the last menstrual period was self-reported. Although we think this is unlikely, it might not have been recalled precisely and have led to an inaccurate estimation of the length of gestation at birth.

We acknowledge that mediation analysis is a sophisticated statistical technique that requires assumptions. The design we used in this study, a prospective cohort, can help us to be confident that pregnancy mental health is a mediator rather than a confounder or an effect-modifier. Nevertheless, the associations between exposure, mediator and the outcome can be confounding or effect modification. Secondly, unmeasured variables including ‘post-traumatic growth’ may have a counter effect on mental health problems that may influence the mediation effect of mental health problems on birthweight. Also, there is the possibility of measurement errors of earthquake experiences and pregnancy mental health because the scales were not locally formally validated against a gold standard comparator for use among pregnant women [28].

The study was powered to detect within-group variation of earthquake experiences on birthweight, but as the earthquake affected the entire country, it was not possible to recruit a comparison group of women who had no experience of the earthquakes. Data were collected about six months after the earthquake. Therefore, the sample did not include women who had already given birth, including those who had premature births. We were told that some women had sought abortions immediately after the earthquakes because they feared that there might have been adverse effects on the foetus. This could lead to underestimates of the impact of the earthquake on infant birthweight. Nevertheless, the study included all eligible women who met inclusion criteria during the study period. The strengths of this study outweigh the limitations and can be generalised with some confidence.

## Implications and conclusions

The study has brought to attention the public health importance of the experiences of earthquakes for women who are pregnant not only for their own health but also because of the impact on their baby’s birthweight. These findings indicate that antenatal CMDs among women should be a focus of post-disaster interventions not only because of the burden [5] imposed on women’s participation but also because of the risk for the growth and development of their foetuses [3, 6].

In our study, a very few women had a low body mass index and preterm birth, which are well-established risk factors of LBW. Despite that our study reported a high prevalence of LBW. This indicates that women’s mental health problems were a key factor for this high prevalence of LBW.

While most participants reported that they received antenatal check-ups, they had a high prevalence of symptoms of antenatal CMDs [11], none of which were detected or treated. Maternal and child health is a national priority in Nepal, and the country has made progress to reduce maternal and child morbidity and mortality in recent decades. However, rates are still very high compared to other countries [1]. In Nepal, there is no specific programs and intervention for the mental health of women in the perinatal period, but this could assist in the reduction of maternal and child morbidity and mortality. These data clearly indicate that specialised consideration of perinatal mental health is a grave need in the post-disaster context in Nepal.

Even though it is challenging to conduct ethical, comprehensive and culturally-sensitive studies after a natural disaster, it is essential to generate evidence to inform effective post-disaster interventions. These data demonstrate that it is feasible to generate robust evidence in this situation.

In conclusion, this study has addressed an important knowledge gap. These findings provide evidence about the importance of women’s antenatal mental health in post-disaster management which is currently missing from the World Health Organization’s guideline “Key Steps for Maternal and Newborn Health Care in Humanitarian Crisis” [37]. The data inform enhancements to existing maternal and child health programs in Nepal and will strengthen the country’s capacity to meet the Sustainable Development Goals [38].

## Supplementary information

**Additional file 1 Supplementary Table 1 (S1)** Model 1 in Sobel test analysis. Multiple linear regression model predicting birthweight from earthquake experiences and other covariates (not including the symptoms of CMDs, the mediator)

**Additional file 2 Supplementary Table 2 (S2)** Model 2 in Sobel test analysis. Multiple linear regression model predicting the symptoms of CMDs from earthquake experiences and other covariates

**Additional file 3 Supplementary Table 3 (S3)** Model 3 in Sobel test analysis. Multiple linear regression model predicting birthweight from the earthquake experiences, the symptoms of CMDs and other covariates

**Additional file 4 Supplementary Table 4 (S4)** Key results of mediation analysis. The Sobel test of the effect of earthquake experiences on birthweight mediated by symptoms of CMDs

**Additional file 5.** Questionnaires. English version of the set of Questionnaires that used to collect primary data

## Abbreviations

CMD: Common mental disorder
EPDS: The Edinburgh Postnatal Depression Scale
LBW: Low birthweight
