# Supplementary material for: Experiences of an earthquake during pregnancy, antenatal mental health and infants’ birthweight in Bhaktapur District, Nepal, 2015: a population-based cohort study
Source: BMC Pregnancy Childbirth. 2020 Jul 20;20:414. doi: 10.1186/s12884-020-03086-5 (PMC7370411; doi:10.1186/s12884-020-03086-5)
Supplement: Supplementary file 1 — Additional file 1 Supplementary Table 1 (S1) Model 1 in Sobel test analysis. Multiple linear regression model predicting birthweight from earthquake experiences and other covariates (not including the symptoms of CMDs, the mediator) [file 12884_2020_3086_MOESM1_ESM.docx]

**Supplementary table 1 Model 1 in Sobel test analysis.**

**Multiple linear regression model predicts birthweight from earthquake experiences and other covariates (not including the symptoms of CMDs, the mediator)**

| Characteristics (N=469) | Coefficient (95% CI) |
| --- | --- |
| Earthquake experiences |  |
| Low experiences (Tertile 1) | Reference |
| Middle/high experiences (Tertile 2 and 3) | 49.03 (-37.68; 135.73) |
| Socio-demographic |  |
| Age (years) | -1.40 (-11.53; 8.74) |
| Body mass index in late pregnancy | 27.24 (16.59; 37.89)*** |
| Education |  |
| No formal/ primary education | Reference |
| Secondary and above education | -60.94 (-177.47; 55.60) |
| Having income-generating work |  |
| No | Reference |
| Yes | 45.95 (-39.34; 131.23) |
| Alcohol consumption |  |
| No | Reference |
| Yes | -80.52 (-179.45; 18.41) |
| Education of partners |  |
| No formal/ primary education | Reference |
| secondary and above education | -25.21 (-160.20; 109.78) |
| Partners’ income-generating work |  |
| No | Reference |
| Yes | 173.85 (19.88; 327.83)* |
| Consume chewing tobacco/smoking by partner |  |
| No | Reference |
| Yes | -79.37 (-170.36; 11.61) |
| Alcohol consumption by partner |  |
| No | Reference |
| Yes | -4.04 (-93.08; 85.00) |
| Household wealth, mean (SD) | 13.59 (-9.05; 36.23) |
| Any lifetime experience of any types of intimate partner violence |  |
| No | Reference |
| Yes | 137.76 (-41.71; 177.20) |
| Reproductive characteristics |  |
| History of pregnancy |  |
| Nulliparous | Reference |
| Two or more pregnancies | 88.42 (-4.17; 181.00) |
| Sex of index foetus |  |
| Boy | Reference |
| Girl | -68.65 (-145.75; 8.48) |
| Length of gestation at baby birth (weeks) | 72.13 (46.46; 97..81)*** |
| Having practical/emotional social support |  |
| No | Reference |
| Yes | 37.65 (-55.39; 130.69) |
| Adjusted R-squared | 0.136 |

Note. N=number; *p<0.05, ***p<0.001
