# Supplementary material for: Experiences of an earthquake during pregnancy, antenatal mental health and infants’ birthweight in Bhaktapur District, Nepal, 2015: a population-based cohort study
Source: BMC Pregnancy Childbirth. 2020 Jul 20;20:414. doi: 10.1186/s12884-020-03086-5 (PMC7370411; doi:10.1186/s12884-020-03086-5)
Supplement: Supplementary file 2 — Additional file 2 Supplementary Table 2 (S2) Model 2 in Sobel test analysis. Multiple linear regression model predicting the symptoms of CMDs from earthquake experiences and other covariates [file 12884_2020_3086_MOESM2_ESM.docx]

**Supplementary table 2 Model 2 in Sobel test analysis**

**Multiple linear regression model predicts the symptoms of CMDs from earthquake experiences and other covariates**

| Characteristics (N=469) | Coefficient (95% CI) |
| --- | --- |
| Earthquake experiences |  |
| Low experiences (Tertile 1) | Reference |
| Middle/high experiences (Tertile 2 and 3) | 1.58 (0.63; 2.53)** |
| Socio-demographic |  |
| Age (years) | 0.97 (-0.01; 0.21) |
| Body mass index in late pregnancy | -0.05 (-0.17; 0.06) |
| Education |  |
| No formal/ primary education | Reference |
| Secondary and above education | -1.07 (-3.07; -0.52)** |
| Having income-generating work |  |
| No | Reference |
| Yes | -2.15 (-3.09; -1.21)*** |
| Alcohol consumption |  |
| No | Reference |
| Yes | 0.57 (-0.51; 1.66) |
| Education of partners |  |
| No formal/ primary education | Reference |
| Secondary and above education | 0.67 (-0.81; 2.15) |
| Partners’ income-generating work |  |
| No | Reference |
| Yes | -2.66 (-3.86;-0.48)* |
| Consume chewing tobacco/smoking by partner |  |
| No | Reference |
| Yes | -0.28 (-1.28; 0.72) |
| Alcohol consumption by partner |  |
| No | Reference |
| Yes | -0.32 (-1.29; 0.66) |
| Household wealth, mean (SD) | 0.20 (0.05; 0.44) |
| Any lifetime experience of any types of intimate partner violence |  |
| No | Reference |
| Yes | 3.58 (2.70; 4.44)*** |
| Reproductive characteristics |  |
| History of pregnancy |  |
| Nulliparous | Reference |
| Two or more pregnancies | -0.27 (-0.75; 1.28) |
| Sex of index foetus |  |
| Boy | Reference |
| Girl | 0.63 (-0.21; 1.48) |
| Length of gestation at baby birth (weeks) | 0.30 (0.01; 0.58)* |
| Having practical/emotional social support |  |
| No | Reference |
| Yes | -1.07 (-2.09; -0.46)* |
| Adjusted R-squared | 0.210 |

Note. N=number; *p<0.05, **p<0.01; ***p<0.001
