# Supplementary material for: Experiences of an earthquake during pregnancy, antenatal mental health and infants’ birthweight in Bhaktapur District, Nepal, 2015: a population-based cohort study
Source: BMC Pregnancy Childbirth. 2020 Jul 20;20:414. doi: 10.1186/s12884-020-03086-5 (PMC7370411; doi:10.1186/s12884-020-03086-5)
Supplement: Supplementary file 3 — Additional file 3 Supplementary Table 3 (S3) Model 3 in Sobel test analysis. Multiple linear regression model predicting birthweight from the earthquake experiences, the symptoms of CMDs and other covariates [file 12884_2020_3086_MOESM3_ESM.docx]

**Supplementary table 3 Model 3 in Sobel test analysis**

**Multiple linear regression model predicts birthweight from the earthquake experiences, the symptoms of CMDs and other covariates**

| Characteristics (N=469) | Coefficient (95% CI) |
| --- | --- |
| EPDS (mean score) | -12.95 (-21.30; -4.60)** |
| Earthquake experiences |  |
| Low experiences (Tertile 1) | Reference |
| Middle/high experiences (Tertile 2 and 3) | 69.52 (-17.41; 156.46) |
| Socio-demographic |  |
| Age (years) | 0.15 (-10.22; 9.93) |
| Body mass index in late pregnancy | 26.56 (15.99; 37.12)*** |
| Education |  |
| No formal/ primary education | Reference |
| Secondary and above education | -84.18 (-200.63; 32.28) |
| Having income-generating work |  |
| No | Reference |
| Yes | 18.06 (-68.34; 104.47) |
| Alcohol consumption |  |
| No | Reference |
| Yes | -73.11 (-11.27; 25.04) |
| Education of partners |  |
| No formal/ primary education | Reference |
| Secondary and above education | -16.54 (-150.43; 117.34) |
| Partners’ income-generating work |  |
| No | Reference |
| Yes | 145.79 (-7.86; 299.45) |
| Consume chewing tobacco/smoking by partner |  |
| No | Reference |
| Yes | -83.03 (-173.22; 7.16) |
| Alcohol consumption by partner |  |
| No | Reference |
| Yes | -8.15 (-96.43; 80.12) |
| Household wealth, mean (SD) | 16.12 (-6.37; 38.61) |
| Any lifetime experience of any types of intimate partner violence |  |
| No | Reference |
| Yes | 84.06 (-0.15; 168.26)* |
| Reproductive characteristics |  |
| History of pregnancy |  |
| Nulliparous | Reference |
| Two or more pregnancies | 191.90 (-0.12; 183.67)* |
| Sex of index foetus |  |
| Boy | Reference |
| Girl | -0.60.43 (-137.03; 16.17) |
| Length of gestation at baby birth (weeks) | 75.97 (50.41; 101.53)*** |
| Having practical/emotional social support |  |
| No | Reference |
| Yes | 23.83 (-68.79; 116.46) |
| Adjusted R-squared | 0.152 |

Note. N=number; **p<0.01, ***p<0.001
