# Supplementary material for: Experiences of an earthquake during pregnancy, antenatal mental health and infants’ birthweight in Bhaktapur District, Nepal, 2015: a population-based cohort study
Source: BMC Pregnancy Childbirth. 2020 Jul 20;20:414. doi: 10.1186/s12884-020-03086-5 (PMC7370411; doi:10.1186/s12884-020-03086-5)
Supplement: Supplementary file 4 — Additional file 4 Supplementary Table 4 (S4) Key results of mediation analysis. The Sobel test of the effect of earthquake experiences on birthweight mediated by symptoms of CMDs [file 12884_2020_3086_MOESM4_ESM.docx]

**Supplementary table 4: Key results of the Sobel test of the effect of earthquake experiences on birthweight mediated by symptoms of CMDs**

| **Findings** | **coefficient** | **Standard error** | **P value** |
| --- | --- | --- | --- |
| EE → CMDs symptoms | 1.58 | 0.48 | 0.001 |
| CMDs symptoms → Birthweight | -12.95 | 4.25 | 0.002 |
| Indirect effect | -20.50 | 9.19 | 0.026 |
| Direct effect | 69.53 | 44.24 | 0.116 |
| Total effect | 49.03 | 44.12 | 0.266 |

Note: EE = earthquake experiences; CMDs = common mental disorders
